# Study Protocol: Global Research Initiative on the Neurophysiology of Schizophrenia (GRINS) project

**DOI:** 10.1186/s12888-024-05882-1

**Published:** 2024-06-10

**Authors:** Jun Wang, Chenguang Jiang, Zhenglin Guo, Sinéad Chapman, Nataliia Kozhemiako, Dimitrios Mylonas, Yi Su, Lin Zhou, Lu Shen, the GRINS Consortium, Yifan Sun, Duxing Li, Zixuan Huang, Jikang Liu, Guanchen Gai, Kai Zou, Zhe Wang, Xiaoman Yu, Limin Chen, Xuezheng Gao, Guoqiang Wang, Wei Zhu, Jess Wang, Lei A. Wang, Yining Wang, Hongliang Zhou, Shen Li, Shengying Qin, Michael Murphy, Shuping Tan, Dara S. Manoach, Robert Stickgold, Hailiang Huang, Zhenhe Zhou, Shaun M. Purcell, Meihua Hall, Steven E. Hyman, Jen Q. Pan

**Affiliations:** 1 The Affiliated Mental Health Center of Jiangnan University, Wuxi Central Rehabilitation Hospital, Wuxi, China; 2 Stanley Center for Psychiatric Research, Broad Institute of MIT and Harvard, Cambridge, United States; 3 Department of Psychiatry, Brigham and Women’s Hospital, Harvard Medical School, Boston, United States; 4 Department of Psychiatry, Massachusetts General Hospital, Harvard Medical School, Boston, United States; 5 Psychiatry Research Center, Beijing Huilongguan Hospital, Peking University Huilongguan Clinical Medical School, Beijing, China; 6 Bio-X Institutes, Shanghai Jiao Tong University, Shanghai, China; 7 Department of Psychiatry, McLean Hospital, Harvard Medical School, Belmont, United States; 8 Beth Israel Deaconess Medical Center, Boston, United States; 9 Department of Psychiatry, Harvard Medical School, Boston, United States; 10 Analytic and Translational Genetics Unit, Massachusetts General Hospital, Harvard Medical School, Boston, United States

**Keywords:** Neurophysiological mechanism, Biomarkers, Sleep EEG, Event-related potential, Brain dynamics, Psychiatric disorders, Schizophrenia, Bipolar disorder

## Abstract

**Background:**

Objective and quantifiable markers are crucial for developing novel therapeutics for mental disorders by 1) stratifying clinically similar patients with different underlying neurobiological deficits and 2) objectively tracking disease trajectory and treatment response. Schizophrenia is often confounded with other psychiatric disorders, especially bipolar disorder, if based on cross-sectional symptoms. Awake and sleep EEG have shown promise in identifying neurophysiological differences as biomarkers for schizophrenia. However, most previous studies, while useful, were conducted in European and American populations, had small sample sizes, and utilized varying analytic methods, limiting comprehensive analyses or generalizability to diverse human populations. Furthermore, the extent to which wake and sleep neurophysiology metrics correlate with each other and with symptom severity or cognitive impairment remains unresolved. Moreover, how these neurophysiological markers compare across psychiatric conditions is not well characterized. The utility of biomarkers in clinical trials and practice would be significantly advanced by well-powered transdiagnostic studies. The Global Research Initiative on the Neurophysiology of Schizophrenia (GRINS) project aims to address these questions through a large, multi-center cohort study involving East Asian populations. To promote transparency and reproducibility, we describe the protocol for the GRINS project.

**Methods:**

The research procedure consists of an initial screening interview followed by three subsequent sessions: an introductory interview, an evaluation visit, and an overnight neurophysiological recording session. Data from multiple domains, including demographic and clinical characteristics, behavioral performance (cognitive tasks, motor sequence tasks), and neurophysiological metrics (both awake and sleep electroencephalography), are collected by research groups specialized in each domain.

**Conclusion:**

Pilot results from the GRINS project demonstrate the feasibility of this study protocol and highlight the importance of such research, as well as its potential to study a broader range of patients with psychiatric conditions. Through GRINS, we are generating a valuable dataset across multiple domains to identify neurophysiological markers of schizophrenia individually and in combination. By applying this protocol to related mental disorders often confounded with each other, we can gather information that offers insight into the neurophysiological characteristics and underlying mechanisms of these severe conditions, informing objective diagnosis, stratification for clinical research, and ultimately, the development of better-targeted treatment matching in the clinic.

**Supplementary Information:**

The online version contains supplementary material available at 10.1186/s12888-024-05882-1.

## Background

Schizophrenia (SCZ) is a severe psychiatric disorder with a lifetime prevalence approximately 1% in the population worldwide, and yet we are limited to symptomatic treatments with a significant side effect burden [1, 2]. Current antipsychotic medications for SCZ ameliorate the positive symptoms in a subset of individuals, with little or even adverse impact on negative or cognitive symptoms [3, 4]. Major challenges in developing effective treatments for SCZ include: 1) the considerable biological heterogeneity across psychotic syndromes, and the variability in longitudinal course of SCZ, which can be indistinguishable from other psychiatric disorders like bipolar disorder (BPD), especially in its early course [5]; 2) not a single region, but interconnected circuit abnormalities across brain regions contribute to functional impairments, consistent with the complex polygenic genetic risk architecture; 3) lacking objective markers to index neurobiological deficits. Currently, treatment is therefore based on clinical symptoms and course with poor insight into underlying mechanisms. Prospects for developing novel therapeutics for SCZ will depend on our ability to 1) better understand disease mechanisms and 2) discover and validate quantitative biomarkers that tag patients’ neurobiological deficits, rather than relying on surface manifestations.

The thalamus is increasingly recognized as a critical node that supports cognitive functions in distributed brain networks, including component processes of attention, memory, executive function, and information processing [6–8]. With its cortical, subcortical, and cerebellar connections, the thalamus coordinates and synchronizes long-range and wide-spread brain-wide activity, and supports cognitive functions as an integrative center that shapes and updates mental representation [8]. Alterations in thalamic morphologies have been reported in both postmortem studies of patients with SCZ and through structural magnetic resonance imaging (MRI) analyses [9, 10]. Functional MRI and diffusion tensor imaging (DTI) have consistently displayed hyperactivity between somatosensory cortex and thalamus and hypoconnectivity between frontal cortex and medial dorsal thalamus in this patient population [11, 12]. Recently, a “trait”-like hyper-connectivity along the long-range cerebello-thalamocortical circuit, specific for SCZ, predicted psychotic conversion in high-risk individuals [13–15]. The emerging critical role of the thalamus in sensory processing and cognition, coupled with abnormal thalamocortical structure and functional connectivity, points to abnormal thalamocortical connectivity as key neurobiological deficits in SCZ [15–17].

The thalamus is involved in regulating states of sleep and wakefulness. Insomnia and other sleep disturbances have been reported in a large proportion of SCZ patients [18–20]. Sleep, in particular non-rapid eye movement (NREM) sleep, is a special lens through which we may quantify intrinsic thalamic and thalamocortical function without confounders from waking behaviors such as active symptoms or altered motivation. Two hallmark features of NREM sleep measured by electroencephalogram (EEG), slow oscillations (SO) and sleep spindles, reflect distinct thalamic and thalamocortical circuits [21, 22]. The slow (1 Hz) neuronal oscillations with large amplitude are generated by cortical neurons and propagated by cortico-thalamo-cortical circuits, while spindles are bursts of oscillatory neural activity (typically between 10 and 16 Hz and ~1 second in duration) arising from reverberant thalamic interaction between thalamic reticular nucleus (TRN) and thalamocortical relay neurons and modulated by thalamocortical connections [23]. The coupling of SOs and spindles mediate information transfer and storage during sleep that underpins NREM’s role in overnight memory consolidation [24]. NREM traits show strong heritability in healthy populations [25], correlate with cognitive performances and provide objective, quantifiable markers of thalamic and thalamocortical functioning in large cohorts [26, 27].

Impaired sleep spindle and SO activities have been reported in individuals with SCZ and their relatives [28, 29], mostly in European and American populations [30], with relatively small sample cohorts (typically, fewer than 30 cases), limiting the power of analyses across diverse functional domains. One relatively large study, comprising 182 cases in Asian population, was reported recently; however, this study did not use high-density EEG and focused on sleep spindles without examining other aspects of sleep neurophysiology such as SO [31]. In addition, it remains unclear how these neurophysiology features are altered along the trajectory of SCZ, and whether there is increased variability at the individual level that can, if it exists, account for the heterogeneity of SCZ. Similarly, several auditory event-related potentials (ERP) measured during wakefulness are impaired in individuals with SCZ, including the auditory steady-state response (ASSR) [32, 33], sensory gating P50 [34, 35], and mismatch negativity (MMN) [36, 37]. These measures also rely on uninterrupted and precise thalamocortical circuit function. However, it is unknown whether these wakefulness indicators represent similar or distinct thalamocortical dysfunctions as those observed during sleep. Most importantly, the similarities and differences of these sleep and wake EEG measures in tracking clinical symptoms, medications response, and cognitive function in SCZ patients remain unclear.

Previous studies have reported alterations in both sleep and wake EEG patterns among patients with BPD and depression [38–42]. However, how altered neurophysiological markers compare across SCZ and other mental disorders has not been well characterized. This is particularly relevant for BPD, a psychiatric condition similarly marked by its complexity, heterogeneity, and high heritability. BPD manifests several symptoms that overlaps with SCZ, including hallucinations, delusions, thought disorder, and others [4, 43] Genome-Wide Association Studies (GWAS) for SCZ and BPD reveal a correlation of around 0.7 [44], and the first penetrant ultra-rare variants associated with BPD, AKAP11, is also shared with SCZ risk [45]. Therefore, investigation of similarities and differences in EEG abnormalities in individuals with SCZ and BPD is warranted.

Given the above evidence, there is an urgent need to thoroughly characterize the neurophysiological abnormities of patients with SCZ and BPD during both sleep and wakefulness, and to elucidate their stability, specificity, heritability and variation with disease trajectory, and implications for non-neurophysiological domains. Research in this area could provide insights into the pathophysiology of SCZ, BPD, and related disorders, and inform the development of innovative therapeutic strategies. Motivated by these, we conceived and launched the Global Research Initiative on the Neurophysiology of Schizophrenia (GRINS) project.

## Methods

### Aims and objectives

The GRINS is an ongoing collaborative research project initiated and primarily funded by the Stanley Center for Psychiatric Research at Broad Institute of MIT and Harvard and its collaborating hospitals. The clinical implementation of GRINS was launched in July 2019 at the Wuxi Mental Health Center (WMHC), also known as the Affiliated Mental Health Center of Jiangnan University, and later expended to Beijing Huilongguan Hospital, an affiliated hospital of Peking University. Both of them are large psychiatric hospitals with more than 1,300 beds. GRINS aims to comprehensively characterize patients with SCZ and other psychiatric disorders by collecting multi-domain phenotypes, including clinical, neurophysiological, genetic, cognitive, and behavioral assessments and measurements in a large representative Chinese cohort. Here, we present the GRINS protocol in detail for transparency and reproducibility. This protocol can serve as a template for large-scale sleep and wake EEG characterization in psychiatric patients, particularly for East Asian population, and as a prototype for optimizing neurophysiological measures with research objectives within this domain in future studies.

### Study design

The GRINS project consists of two phases. In the first phase, we have employed a cross-sectional design to 1) verify and characterize previously reported sleep and wake EEG metrics; 2) to determine the extent of their correlations and interactions; 3) to examine the relationship between EEG metrics and clinical and cognitive measures; 4) to explore the variability of these measures within and across cases and controls; and 5) to identify potential new neurophysiological biomarkers for SCZ, individually or in combination, with a special emphasis on NREM sleep EEG metrics, such as the Chirp of sleep spindle [46]. In the second phase, in addition to the cross-sectional design, we are adding a longitudinal follow-up design and are expanding to include BPD patients. Main objectives of this phase are to ascertain: 1) the progression of sleep and wake EEG metrics with the disease trajectory, and 2) the specificity of these indicators in SCZ as compared to BPD.

**Fig. 1:**
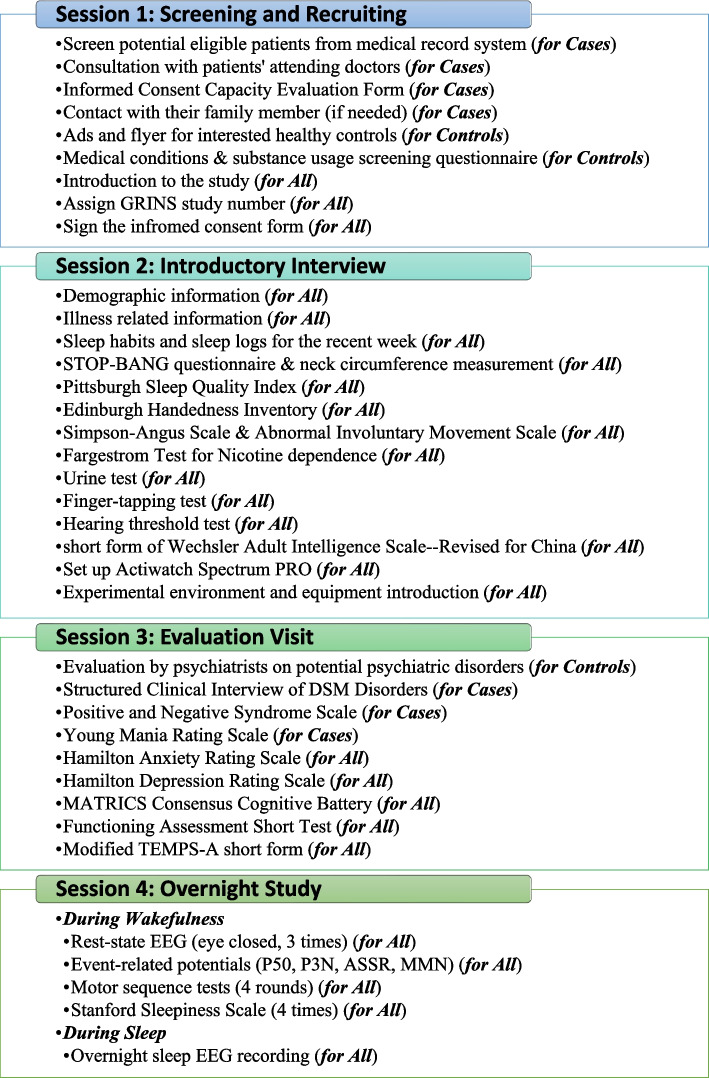
Overall procedure of the GRINS

The GRINS team includes clinical psychiatrists, scientists specializing in neurobiology, electrophysiology, and genetics, computational scientists, and information technologist, as well as project management professionals, in both the US and China. Dedicated assessment teams are organized for clinical evaluations and neuropsychological tests in this study (detail in section Study Management). The overall procedure for this protocol is comprised of four sessions, as depicted in Fig. 1.

The study protocol of GRINS has received approval from the Harvard TH Chan School of Public Health Office of Human Research Administration (IRB18-0058), and the Institutional Review Boards of WMHC (WXMHCIRB2018LLKY003), Beijing Huilongguan Hospital (2023-07-KE), and Shanghai Jiao Tong University (M16035). Our research activities strictly adhere to the principles of the Declaration of Helsinki. All participants voluntarily take part and written informed consents are obtained from each prior to investigation. Participants are compensated for taking part in the GRINS based on local standard of living. Control subjects and outpatients are additionally compensated for transportation.

### Study population

SCZ and BPD patients are recruited from psychiatric hospitals such as WMHC, and HCs are recruited from local community residents. In our research, we do not intervene in the treatment regimens of patients; all patient care and therapeutic approaches remain under the guidance and discretion of their clinical care providers.

The inclusion criteria for the SCZ group are: a) patients clinically diagnosed with “schizophrenia” based on the Diagnostic and Statistical Manual of Mental Disorders (DSM-5) criteria; b) an age range of 18-45 years and an intelligence quotient (IQ) of no less than 70; c) clinical stability sufficient to undergo an overnight sleep EEG study; and d) a finger-tapping test requirement of typing a total of ≥ 24 sequences of “1-2-3-4” during two 30-second trials with the left hand (the right hand may be used only in case of injury). The inclusion criteria for the BPD group are: a) patients clinically diagnosed with “bipolar disorder” based on the DSM-5 criteria; and the b) to d) criteria are the same as for the SCZ group. The inclusion criteria for the HC group include a) no current or previous diagnosis of any mental disorder, including mental retardation and dementia, and no family history of psychiatric illness; b) age and gender matching with the SCZ group; and c) the same prerequisites as criteria b) to d) for the SCZ group.

The shared exclusion criteria for all three groups (SCZ, BPD, and HC) are as follows: a) being outside the age range of 18-45; b) medical diagnosis of any sleep disorders such as insomnia, hypersomnia, and periodic limb movement disorder, or self-reported frequent difficulty in falling asleep and waking up easily during the night, or a STOP-BANG score of 4 or above, indicating a high risk of obstructive sleep apnea hypopnea syndrome [47]; c) use of barbiturate medications; d) drug abuse within the past 6 months, or a positive urine test at enrollment; e) presence of significant medical or neurological illness like epilepsy, or history of brain injury with loss of consciousness over 15 minutes; f) having received electroconvulsive therapy (ECT) within the past 6 months; g) a hearing threshold above 45dB at 1000Hz as determined by an audiometer; h) current pregnancy or breastfeeding; and j) legal and/or mental incompetence. Additional exclusion criteria for the case groups (SCZ and BPD) include mental disorders triggered by substances or other medical conditions.

Across the two phases, the project aims to recruit approximately 200 patients with SCZ and 100 patients with BPD, covering a range from early to chronic stage. Additionally, 200 age- and sex-matched healthy controls (HC) will be included. It is anticipated that 100 to 150 participants will be willing to engage in and complete a follow-up study in the second phase with an interval of around one year. Sample size considerations indicate that a total sample of *N*=170 can detect a modest effect size d=0.43 with 80% power (5% type I error rate). In the pilot study, the effect size of significantly impaired neurophysiological markers in SCZ range between 0.54 and more than 1.5 [48]. We estimate that our cohort size should be able to address the question that we aim to answer.

### Study procedure

As illustrated in Fig. 1, the study procedure is divided in to four sessions, namely, screening and recruiting, introductory interview, evaluation visit, and overnight study, as described below. During the follow-up period, participants will undergo the same procedure as above, ensuring consistency in data collection and allowing for subsequent longitudinal comparisons.

#### Session 1: screening and recruiting

As the study protocol requires several sessions including an overnight visit, both SCZ and BPD patients are primarily recruited from inpatient units to minimize the risk of dropout. Potential eligible patients are identified via reviewing the medical records and confirmed with their attending physician who approve patient’s clinical stability and suitability for participation. Those who are deemed suitable are then approached in person by the GRINS research team. Patients who agree to participate in the study complete the study-specific “Informed Consent Capacity Evaluation Form” (see Supplementary Material 1), to evaluate whether they fully understand the study’s procedures, potential risks and benefits, as well as their rights and obligations in participating.

HCs are recruited through advertisements. Additionally, a social media mobile application named WeChat is authorized and utilized to recruit control subjects. Eligibility screening for individuals interested in participating is conducted through the “GRINS Controls Screening Questionnaire” (see Supplementary Material 2) via phone, WeChat, or in-person.

Upon successfully passing the screening, participants (both patients and control subjects) are considered formally enrolled and assigned a unique GRINS study number. The numbers are sequentially coded for all participants and blind to group membership. Informed consent is obtained from each participant before initiating the Introductory Interview. Following the completion of the consent form, blood samples from the participants are collected.

#### Session 2: introductory interview

During this session, participants are required to complete specific questionnaires and tests such as the STOP-BANG questionnaire, finger-tapping test, hearing threshold test, and urine test. The short form of the Wechsler Adult Intelligence Scale—Revised for China (WAIS-RC-S), containing four subtests (information, similarities, block design, picture completion), is used to calculate the IQ of each participant. An “Enrollment Checklist” (see Supplementary Material 3) is used to further determine participant’s eligibility based on collected information.

Demographic and clinical information is collected. Details of the medications that participants are currently taking are recorded, specifying medication names, categories, and dosages. Such records facilitate subsequent analyses related to medication use. Information on handedness, extrapyramidal symptoms, tardive dyskinesia, nicotine dependence, as well as sleep habits and quality are also collected (Table 1 and Fig. 1). At the end of this session, participants are equipped with the Actiwatch Spectrum PRO (Phillips), a compact device designed for tracking daily activity and sleep patterns. They are instructed to wear this device continuously throughout the study, for a minimum duration of three days and nights. Additionally, participants are introduced to the sleep medicine center, which aims at familiarizing them with the study environment and procedures.

**Table 1:** Instruments used in GRINS

**Instrument**	**Description**	**In Session**	**Cases**	**Controls**
AIMS	A 12-step assessment for tardive dyskinesia	2	×	×
EHI-SF	A 4-question inventory to determine dominant hand	2	×	×
FTND	A 7-item questionnaire to screen physical nicotine dependence	2	×	×
PSQI	A 9-item (18 questions) questionnaire to quantify recent sleep quality	2	×	×
SAS	A 11-item questionnaire to assess extrapyramidal symptoms	2	×	×
SHQ	A customized questionnaire designed to screen for sleep disorders and assess participants' typical sleep habits	2	×	×
STOP-BANG	An 8-item questionnaire to screen for obstructive sleep apnea	2	×	×
TEQ	A customized questionnaire designed to identify potential factors affecting the finger tap test	2	×	×
WAIS-RC-S	A Short-form Wechsler Adult Intelligence Scale with four subtests for quick intelligence quotient assessment in adults	2	×	×
FAST	A 24-item tool to evaluate functional impairment, covering autonomy, occupational functioning, cognitive abilities, financial issues, interpersonal relationships, and leisure time.	3	×	×
HAMA	A 14-item clinical assessment tool for evaluating the severity of anxiety symptoms	3	×	×
HAMD	A 24-item clinical assessment tool for evaluating the severity of depressive symptoms	3	×	×
MCCB	A standard tool comprising 10 subtests designed to comprehensively evaluate 7 cognitive domains	3	×	×
PANSS	A 30-item questionnaire to assess the severity of different aspects of mental symptoms	3	×	/
SCID	A semi-structured interview guide featuring several modules for diagnosing DSM mental disorders	3	×	/
TEMPS-A	A self-report questionnaire that assesses various temperamental dimensions, including cyclothymic, depressive, hyperthymic, irritable, and anxious traits	3	×	×
YRMS	An 11-item clinical assessment tool for evaluating the severity of manic symptoms	3	×	/
SSS	A self-report questionnaire designed to measure a person's level of sleepiness	4	×	×

*AIMS* Abnormal Involuntary Movement Scale, *EHI-SF* Edinburgh handedness inventory-Short Form, *FAST* Functioning Assessment Short Test, *FTND* Fagerstrom Test for Nicotine Dependence, *HAMA* Hamilton Anxiety Rating Scale, *HAMD* Hamilton Depression Rating Scale, *M.I.N.I.* Mini International Neuropsychiatric Interview, *MCCB* MATRICS Consensus Cognitive Battery, *PANSS* Positive and Negative Syndrome Scale, *PSQI* Pittsburgh Sleep Quality Index, *SAS* Simpson-Angus scale, *SCID* Structured Clinical Interview for DSM Disorders, *SHQ* Sleep Habit Questionnaire; SSS: Stanford Sleepiness Scale, *TEMPS-A* short form of the Temperament Evaluation of Memphis, Pisa, Paris and San Diego-autoquestionnaire, *TEQ* Typing Experience Questionnaire, *WAIS-RC-S* Short form of the Wechsler Adult Intelligence Scale—revised for China, *YMRS* Young Mania Rating Scale

#### Session 3: evaluation visit

This session involves a series of clinical assessments and neuropsychological testing conducted by trained psychiatrists specialized in each domain. Patients’ diagnosis is confirmed through the Structured Clinical Interview for DSM Disorders (SCID). Controls’ medical histories are examined by a psychiatrist to rule out psychiatric disorders. Severity of psychiatric symptoms is assessed using the Positive and Negative Syndrome Scale (PANSS), and cognitive functions are evaluated using the MATRICS Consensus Cognitive Battery (MCCB). Consistent with the inclusion of patients with BPD in the second phase, symptom assessments are also expanded to incorporate the Young Mania Rating Scale (YMRS), the Hamilton Depression Rating Scale (HAMD), and the Hamilton Anxiety Rating Scale (HAMA). Additionally, the Functioning Assessment Short Test (FAST) is adopted to evaluate the functional impairment. A modified short form of the Temperament Evaluation of Memphis, Pisa, Paris and San Diego-autoquestionnaire (TEMPS-A) is used to measure participants' temperamental characteristics. Table 1 summarizes the instruments used.

#### Session 4: overnight study

**Fig. 2:**
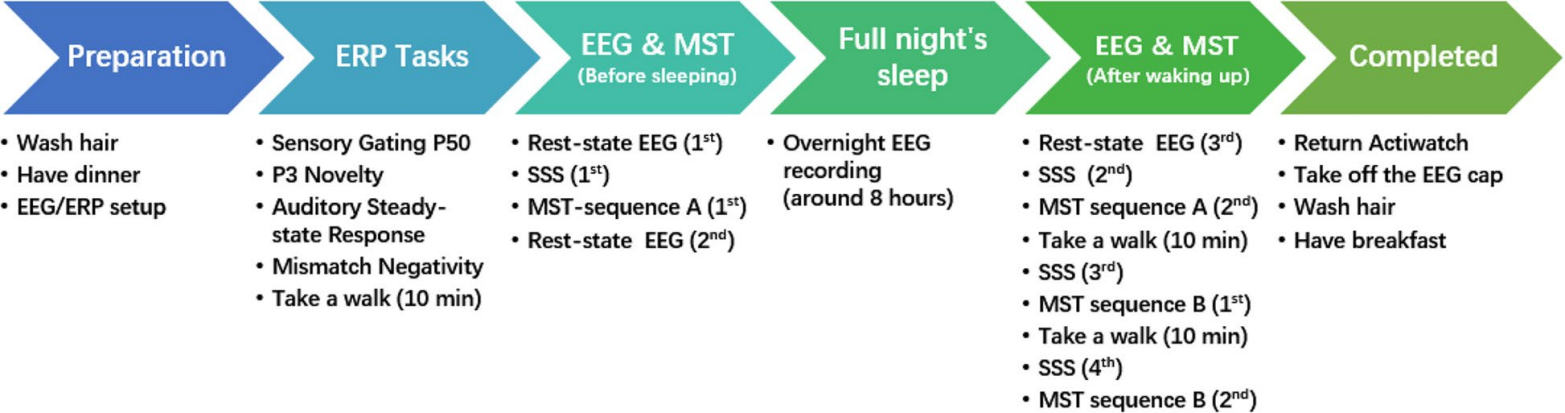
Flow chart of tasks and processes during the overnight study session. ERP: event-related potentials; MST: motor sequence tests; SSS: Stanford Sleepiness Scale

This session includes EEG recordings in four awake ERP recording paradigms, three periods of wake resting state with eyes closed, an overnight sleep, and four rounds of motor sequence test (MST), as depicted in Fig. 2. Briefly, the ERP tasks are conducted first, followed by a resting state EEG and then one round of MST. Another resting state EEG is performed before sleep, followed by the overnight sleep EEG recording. Upon waking the next morning, participants undergo another resting state EEG and three rounds of MSTs. The Stanford Sleepiness Scale (SSS) is administered to detect drowsiness, and there are three 10-minute ‘take a walk’ breaks following ERP or MST sessions to maintain alertness.

### Data collection

Comprehensive data are collected for each participant during the aforementioned sessions. With the exception of data from EEG/ERP, MST, and Actiwatch, all other data are documented in an electronic report form (ERF) application tailor-made for the GRINS project. This application incorporates built-in validation rules to minimize entry errors. Additionally, the timestamps of data entries are logged, offering supplementary insights for both study management and quality control.

We use the BrainAmp Standard Amplifiers (Brain Products GmbH, Germany) to record participants’ brain electrical activity signals during ERP tasks, wake resting state, and sleep period, at a sampling rate of 500 Hz. A 64-channel BrainCap (EASYCAP, GmbH) with flat electrodes is employed, featuring a customized electrode layout and channel assignment suitable for overnight sleep recordings (see Supplementary Material 4). The electrode placed under the left clavicle served as the ground, and the one placed on the forehead as a recording reference. The vertical electrooculogram (VEOG) is monitored by the electrode placed at the lower orbit of the left eye, and the horizontal electrooculogram (HEOG) recording electrodes are placed 1 cm from the outer canthus of both eyes. Bilateral mastoid electrodes are set up for subsequent EEG data preprocessing, if appropriate. Electromyogram (EMG) is recorded by placing two electrodes on the skin overlying the masseter muscle on both sides of the jaw. During the recording, the electrode impedances are kept below 10 kOhm. Different paradigms are described as below:

#### Part 1. Wake ERP tasks

A total of four well-established ERP tasks are run sequentially: sensory gating P50 (P50), P3 novelty (P3N), auditory steady state response (ASSR), and mismatch negativity (MMN). Stimuli are either displayed on the screen or presented through foam insert earphones using the Presentation software (Neurobehavioral Systems, NBS). All ERP data preprocessing is performed using BrainVision Analyzer (Brain Products, Germany), following established methods as previously reported [48–51]. Since these paradigms have been extensively utilized and reported, we provide only a brief description here.

The P50 paradigm measures auditory sensory gating function. Paired identical dual-click stimuli (S1 and S2) are presented in this paradigm. Each click is 5 ms long (2 ms rise/fall period), and a pair of clicks is presented with a 500 ms inter-click interval. The inter-trial interval is 10 seconds. There is a total of 160 click pairs averagely divided into 4 blocks that are separated by a 1-minute break. During this task, participants are asked to stay awake.

The P3N paradigm consists of 400 binaural auditory tones at 80 dB: 76% standard tones (lasting for 50 ms at 1000 Hz), 12% target tones (lasting for 50 ms at 1500 Hz), and 12% novelty tones presented as either sounds of running water or bird chirping. The three types of stimuli occur in a randomized sequence, with an inter-stimulus interval of 1000ms. Participants are instructed to click a mouse as quickly as possible when they hear the target stimuli but not respond towards both standard and novelty tones. This paradigm generates a number of response components including N1, P2, P3a, and P3b.

The ASSR paradigm elicits frequency specific oscillatory responses at 20 Hz, 30 Hz, and 40 Hz. For each frequency stimulation (in a randomized order), there are 150 trains of 1 ms white noise clicks with a 500 ms duration and a stimulus onset asynchrony of 1100 ms at corresponding rate. Participants are required to stay awake.

The MMN paradigm measures auditory processing and sensory memory. The paradigm consists of a total of 1200 stimuli, of which 85% are standard pure tones (1000 Hz, 100 ms) and 15% are deviant pure tones (1000 Hz, 150 ms), with an inter-stimulus interval of 200 ms. In this task, participants are instructed to watch a peaceful, soundless cartoon video clip. MMN is obtained by subtracting the ERP evoked by standard sounds from those evoked by deviant ones.

#### Part 2. Rest-state EEG recording

Rest-state EEG measures the spontaneous brain activity during quiet rest. There are a total of three rounds of rest-state EEG recording: one before and one after the first MST prior to the overnight sleep EEG, and then another before the second MST the following morning. Participants are instructed to relax, minimize movements (such as blinking or swallowing) as much as possible, and keep their eyes closed throughout these recordings. Each EEG recording lasts for no less than 5 minutes. During this time, participants are instructed to stay awake.

#### Part 3. Overnight sleep EEG recording

Overnight sleep EEG measures spontaneous brain activities across different stage of sleep. After completing all tasks required before the overnight sleep EEG recording session, patients take their prescribed medications. The sleep EEG recording starts when participants go to bed. A researcher in an adjacent room monitors the overnight sleep recordings. If participants need to use the bathroom during the night, they can ring a bell, prompting the on-duty researcher to pause and resume the EEG recording and assist the participants as needed.

We use Luna (http://zzz.bwh.harvard.edu/luna/), developed by a member of GRINS team (SP), to preprocess both resting state EEG and sleep EEG data. For a detailed methodology, please see references [48] and [52].

#### Motor sequence tests

MST is employed following established method [53–55] to assess individual’s motor memory status. Briefly, participants are instructed to tap a designated sequence of numbers (i.e., 41324 or 23142) on a four-key pad as quickly and accurately as possible (Supplementary Material 5). Each sequence undergoes a training and a testing round. Sequence A is trained pre-sleep (Round 1) and tested post-sleep (Round 2), while Sequence B is trained (Round 3) and tested (Round 4) the following morning, with a 10-minute rest in between. During training and testing, participants are asked to type using their left hand for 30 seconds per trial, followed by a 30-second inter-trial rest. There are 12 trials in Round 1 to 3, and 6 trials in Round 4. Prior to each round, there is a warm-up session during which participants are instructed to quickly click on keys labeled 3 and 4 using their right hand. Participants are prompted by screen color changes and beeps during tests. A reward is offered for total correct sequences to motivate performance. The primary indicators of interest include typing speed, accuracy, pattern consistency, and learning rate, particularly focusing on pre- and post-sleep performance.

### Statistical analysis plan

In cross-sectional data analysis, our primary focus is on examining group differences and intra-group variation. We detailed the statistical methodology in our report on the results from the wave 1 data of the cross-sectional study of GRINS in 2022 [48]. For subsequent analyses, we intend to adopt similar methodologies with an emphasize on the trends or patterns of change observed over time within groups. In brief, demographic data between groups will be analyzed using appropriate methods, such as the F-test or the Chi-square test. For any variable that shows a significant group-difference or is considered relevant to our indicators of interest based on previous evidence (like age and sex), it will be used as a covariate in subsequent statistical comparisons. Based on the nature of the data, logistic regression or linear regression, incorporating important covariates, will be used to assess group differences in variables of interest, explore associations across variables, and develop prediction models for diseases. For longitudinal analyses, we will use linear mixed models to account for correlated repeated measurements. We will test for missing observations that are not missing completely at random (MCAR), especially if missing status correlates with other measures. Cluster-based permutation testing is employed to control for false positive rates arising from the multiplicity of tests across different channels and frequencies. We will also conduct sensitivity analyses to address potential confounders, such as age and sex, with special consideration given to assessing the impact of medications. In addition, we will examine subgroup differences among medications when there are 10 or more patients using a particular medication. Individually, all measures will be transformed to approximate normality as needed (e.g., applying a log-transform to spectral power metrics), and statistical outliers will be removed prior to the main analyses.

### Study management

A specialized research team at WMHC is assembled for the execution of the GRINS project, organized into five groups due to the extensive range of clinical evaluations and neuropsychological tests involved. Each group is responsible for assessing different aspects: SCID, PANSS, Cognition (MCCB), mood status (HAMA, HAMD, and YMRS), and other areas. Prior to the formal launch, every team member underwent relevant training tailored to their specific roles and responsibilities within the study. All assessors received hands-on training from experts at Beijing Huilongguan Hospital and the Second Xiangya Hospital to ensure inter-rater reliability.

Weekly online meetings are conducted to monitor the progress of the project, attended by the principal investigators from both the US and China, the project manager, the coordinator, and researchers from GRINS. During these sessions, we discuss weekly research updates, assign upcoming tasks, report on data quality control results, and more. Feedback from participants and research staff is regularly gathered to identify areas for improvement. The Office of Regulatory Affairs & Research Compliance of Harvard TH Chan School and the Institutional Review Board of WMHC also provide additional onsite reviews to help address deficiencies and promote better organization and implementation.

Data are routinely backed up to secure servers. Security of and access to data have been designed to protect group membership (blind) and data breaching. For instance, members of the MCCB evaluation team can view participants’ basic demographic information and have full access to the MCCB section, but they cannot access other data. In contrast, members of the analysis team are not permitted to access grouping information until the data analysis at the individual level is finalized.

Preliminary data quality control checks are conducted weekly, focusing on data integrity, consistency, and reliability. These checks utilize both software scripts and manual reviews. For instance, an age validation procedure involves juxtaposing the age input into the electronic system with the age deduced from the participant’s date of birth, facilitating the identification and correction of discrepancies. The power spectral density of each sleep EEG is graphed to inspect for any signs of harmonic interference. Results stemming from these quality control measures are collectively reviewed and deliberated upon during our regular online meetings, as abovementioned.

## Discussion

The GRINS project endeavors to comprehensively characterize the neurophysiological abnormities of SCZ and other psychiatric illnesses, primarily focuses on the thalamocortical system. Dysfunction in the thalamus and its cortical connections is believed to be related to the dysregulation of dopamine and glutamate neurotransmission, both of which are implicated in the pathophysiology of SCZ [56]. The thalamocortical system plays a pivotal role in the integration of sensory information and in the processing of higher cognitive functions such as perception, attention, and memory. These functionalities are intrinsically linked to various symptoms of SCZ, including hallucinations, delusions, and cognitive deficits [57–59]. Previous research also suggests that thalamo-cortical circuits are responsible for generating slow-wave activity and NREM sleep spindles [53, 60], while abnormalities in sleep architecture and neurophysiology have been repeatedly reported in individuals with SCZ [18–20] and may correlate with their clinical symptoms [31]. By integrating and conducting in-depth analysis of multidimensional neurophysiological metrics associated with thalamocortical system, it becomes possible to ascertain the roles of, and further clarify the mechanisms behind, potential neurophysiological markers for SCZ.

Currently, the GRINS project has successfully enrolled over 300 participants, and data collection is ongoing. Analysis of the pilot study dataset, comprising 72 cases and 58 controls, indicated excellent data quality and validated key wake and sleep EEG abnormalities observed in SCZ populations from Europe and America [48]. This suggests that the study protocol can capture the key neurophysiological parameters of SCZ. With data from additional participants and longitudinal follow-up studies, we will be able to better understand the significance of neurophysiological markers for SCZ and the underlying mechanisms.

Conducting a time-consuming overnight study like GRINS on patients with psychiatric disorders poses unique challenges. The experience and strategies we have accumulated thus far may offer insights for future research in this domain: Firstly, we endeavored to streamline the study process, ensuring clarity and ease of understanding. To minimize potential errors during critical steps, we established standardized operating procedures and guidelines for research team members. These include instructions on correctly positioning and setting the BrainCap, naming conventions for various data sets, and precautions for the overnight study. Also, we created multiple groups for lengthy clinical assessments. In this setup, every research staff can easily understand their roles and responsibilities, thereby enhancing the efficiency and accuracy of the study.

Various measures are implemented to prevent nervousness, fatigue, and resistance among participants, thereby enhancing their cooperation. We educate them about EEG basics and safety prior to the overnight study, with a special focus on demonstrating the electrode cap to alleviate any potential concerns. A pre-visit to the sleep center is arranged to reduce new-environment anxiety, aiming to minimize the “first-night effect” [61]. During the overnight study, we intersperse active tasks within passive paradigms and schedule breaks between tasks to maintain alertness and minimize drowsiness. Performance-based rewards are offered to boost focus and concentration. Additionally, snacks are prepared for participants in case of nighttime hunger.

In our study, we uphold several key ethical considerations: We avoid interfering with any patient’s clinical treatment and ensure that patients fully understand the study by utilizing an “Informed Consent Capacity Evaluation Form”. In the context of Chinese society, where family often plays a crucial role in decision-making, we also inquire about the patient’s willingness to involve family members in the decision-making process. If a family member advises against participation, we respect this decision and ensure it doesn’t impact the patient’s medical services. Additionally, we consult with the patient’s attending physicians to confirm clinical stability for the overnight study and, when necessary, station security guards near the sleep laboratory for added safety.

It should be noted that while necessary criteria are used to ensure participant safety and streamline the study process, certain limitations may be introduced. For example, the recruitment of clinically stable patients may limit the representativeness of the study sample. In addition, patients are primarily recruited from inpatient unit. While this minimizes the risk of dropout, the hospital-imposed routine may not reflect naturalistic sleep/wake rhythms and circadian patterns. At the same time, allowing the joint use of benzodiazepines and mood stabilizers may affect certain aspects of sleep EEG [62], although group differences between SCZ and controls still persisted even after statistically controlling for the effects of medication in our pilot results [48]. Last but not the least, the study design involved a single overnight EEG recording session. Despite the fact that we did not observe the “first-night effect” in previous studies [62, 63] and our efforts to mitigate it in the current study, its potential influence on the results may not be completely disregarded.

## Conclusion

The GRINS project is dedicated to deepening our understanding of the neurophysiological abnormalities associated with SCZ, by employing a large sample size across multimodal measurements. The pilot results successfully validated key neurophysiological phenotypes of SCZ in the Chinese population and affirmed the project’s feasibility. Through GRINS, we are generating a valuable multi-dimensional dataset with the potential to uncover neurophysiological biomarkers for SCZ and other psychiatric disorders, and their underlying mechanisms. This protocol offers the flexibility to be applied across psychiatric disorders and to integrate additional biological research methodologies and goals. Such discoveries may inform objective diagnoses, patient stratification, prognostic predictions, and the development of innovative therapeutic strategies for these conditions.

### Supplementary Information

Supplementary Material 1. 

## Data Availability

All data will be shared with the research members of the GRINS consortium as they become available. Anonymized individual-level data from the pilot study is accessible in the Dryad archive (10.5061/dryad.j0zpc86h4). Additional anonymized, individual-level data will be made available to researchers outside the GRINS consortium upon request following publication. Please direct inquiries to JP at jpan@broadinstitute.org.

## Abbreviations

AIMS: Abnormal Involuntary Movement Scale
ASSR: Auditory steady-state response
BPD: Bipolar disorder
BROAD: Stanley Center for Psychiatric Research at Broad Institute of MIT and Harvard
DSM: Diagnostic and Statistical Manual of Mental Disorders
DTI: Diffusion tensor imaging
ECT: Electroconvulsive therapy
EEG: Electroencephalogram
EHI-SF: Edinburgh Handedness Inventory-Short Form
EMG: Electromyogram
ERP: Event-related potentials
FAST: Functioning Assessment Short Test
FTND: Fagerstrom test for nicotine dependence
GEV: global explained variance
GFP: global mean field power
GRINS: Global Research Initiative on the Neurophysiology of Schizophrenia
GWAS: Genome-Wide Association Studies
HAMA: Hamilton Anxiety Rating Scale
HAMD: Hamilton Depression Rating Scale
HEOG: Horizontal electrooculogram
IQ: Intelligence quotient
MCAR: Missing completely at random
MCCB: MATRICS Consensus Cognitive Battery
MMN: Mismatch negativity
MRI: Magnetic resonance imaging
MST: Motor sequence test
NREM: Non-rapid eye movement
P3N: P3 novelty
PANSS: Positive and Negative Syndrome Scale
PSQI: Pittsburgh Sleep Quality Index
SCID: Structured Clinical Interview for DSM Disorders
SCZ: Schizophrenia
SO: Slow oscillations
SSS: Stanford Sleepiness Scale
TEMPS-A: Temperament Evaluation of Memphis, Pisa, Paris and San Diego-autoquestionnaire
TRN: Thalamic reticular nucleus
VEOG: Vertical electrooculogram
WAIS-RC-S: Short form of the Wechsler Adult Intelligence Scale—revised for China
WMHC: Wuxi Mental Health Center
YMRS: Young Mania Rating Scale
