# Supplementary material for: Study Protocol: Global Research Initiative on the Neurophysiology of Schizophrenia (GRINS) project
Source: BMC Psychiatry. 2024 Jun 10;24:433. doi: 10.1186/s12888-024-05882-1 (PMC11165775; doi:10.1186/s12888-024-05882-1)

**Study Protocol: Global Research Initiative on the Neurophysiology of Schizophrenia (GRINS) project**

**Supplementary Materials**

**Supplementary Material 1:** The Informed Consent Capacity Evaluation Form for the GRINS Project.

**Supplementary Material 2:** The Controls Screening Questionnaire for the GRINS Project.

**Supplementary Material 3:** The Enrollment Checklist for the GRINS Project.

**Supplementary Material 4:** The Electrode Layout and Channel Assignment for the Customized 64-Channel BrainCap in the GRINS Project.

**Supplementary Material 5:** Schematics of the Finger-Tapping Test and Motor Sequence Tests.

**Supplementary Material 1: The Informed Consent Capacity Evaluation Form for the GRINS Project.**

**Informed Consent Capacity Evaluation Form**

1) Do you know what illness is studied in this research project?

2) If you don’t want to, do you have to be in this study?

3) If you do not want to be in this study, will you suffer any adverse effects (for example lose your benefits or be treated differently by doctors)?

4) If you want to, when could you quit the study?

5) How long will you be invited back for follow-up assessments?

6) If you have any questions about this study, who can you ask? How can you reach them?

7) How much money will you receive for completing the study?

8) If you don’t want to be in this study, will you still be able to get treatment for your illness?

9) What will you do in this study?

Conducting clinician Date

**Supplementary Material 2: The Controls Screening Questionnaire for the GRINS Project.**

**GRINS Controls Screening Questionnaire**

Name:

Date:

**Intro**

I will need to ask you several questions about your personal history to be sure that you are eligible for the study. This takes a couple of minutes.

Basic Information

What is your age? _______________________

D.O.B. ______________________

**Medical Information**

1. Have you ever been diagnosed for sleep disorders (APNEA, narcolepsy, insomnia) by a physician?
2. Have you ever had insomnia (frequently wake up, often takes more than 1 hour to sleep, and sleep <5 hours always)?
3. Have you ever had head trauma or injury with a loss of consciousness that is more than 15 minutes?
4. Are you in a lot of pain, or are you very sick now? Have you had to go to the emergency room in the past three months?
5. Have you ever had a seizure? Have you ever been diagnosed as epilepsy?
6. Do you have any problems with your vision or hearing?
7. Are you currently taking antidepressants (SSRIs, SNRIs, SARIs, TCAs, MAOIs, Buproprion, Buspirone), anticonvulsants (lithium, lamotrigine, carbamazepine, oxcarbamazepine, topiramate), barbiturates, benzodiazepines. Z-drugs (zolpidem, eszopiclone)
8. are you currently taking anticholinergics (benztropine, diphenhydramine), or adrenergic agents (clonidine, propranolol, prazosin etc.]
9. Do you have any allergies (food, drug, latex?)
10. Have you ever seen a psychiatrist, therapist, or counselor (ie. school)?
11. Have you ever had any psychiatric conditions, such as depression, any type of anxiety, psychosis, bipolar disorder, schizophrenia, OCD, ADD/ADHD, eating disorder, anorexia, bulimia, autism?
12. Do any of your parents, siblings, or children have a history of psychiatric illness, depression, anxiety, psychosis, bipolar disorder, schizophrenia, OCD, ADD/ADHD, eating disorder, anorexia, bulimia, autism, problems with alcohol or drugs?
13. Are you currently taking barbiturates?
14. Are you currently taking benzodiazepines?

**Substance Use**

1. Do you smoke cigarettes? 1/day, 10/day, 1 pack/day
2. Do you drink alcohol regularly?
3. Any drug abuse or addiction?

Well, this all sounds fine. We would like to have you participate if you are still interested.

Appointment Date & Time________________________________________________

I’ll also call you a day or so in advance of your visit just to confirm our plans and see if you have any additional questions. I’ll need your email address and telephone number to reach you. Is this the best telephone number to reach you? What is a good time to call when I confirm your appointment? If you’re not there, is it okay to leave a message with someone or on your answering machine. Thank you very much for your time. I look forward to meeting you.

Name: ______________________________________________________________

Email: __________________________

Telephone: (H) _________________________ (C) _________________________

Can leave message: Yes_____ No_____

Date_______________________

Subject ID#______________________________________________________________

**Supplementary Material 3: The Enrollment Checklist for the GRINS Project.**

**Enrollment Checklist**

**1. Eligibility Checklist**

Participant Name: Participant ID: Date:

| **Inclusion Criteria** | | |
| --- | --- | --- |
| Within age range of 18-45 years old | Yes | No |
| Common Knowledge Test within 2 standard deviations from the population norm | Yes | No |
| Finger-tapping test meets typing a total of ≥ 24 sequences of “1-2-3-4” during two 30s trials with the left hand (only in case of injury can right hand be used) | Yes | No |
| Clinically diagnosed with schizophrenia or bipolar disorder based on DSM-5 criteria (***for Cases***) | Yes | No |
| Clinical stability sufficient to undergo an overnight sleep EEG study (***for Cases***) | Yes | No |
| No current or historical diagnosis of any type of mental disorder (***for Controls***) | Yes | No |
| Age and sex matched to the schizophrenia group (***for Controls***) | Yes | No |
| ***If you checked NO to any of the above questions, STOP. This subject is not eligible!*** | | |
| **Exclusion Criteria** | | |
| Outside the age range of 18-45 | Yes | No |
| With diagnosed sleep disorders, or self-reported severe sleep difficulties | Yes | No |
| STOP-BANG score of 4 or above | Yes | No |
| Are taking barbiturate medications | Yes | No |
| Had drug abuse within the past 6 months, or urine test positive | Yes | No |
| Significant medical or neurological illness | Yes | No |
| Past brain injury with lost conscious over 15 minutes | Yes | No |
| Received ECT within the past 6 months | Yes | No |
| Hearing impairment (hearing threshold above 45dB at 1000Hz) | Yes | No |
| Pregnancy or breastfeeding | Yes | No |
| Legally and/or mentally incompetent | Yes | No |
| Psychotic episodes attributable to the physiological effects of substances or other medical conditions (***for Cases***) | Yes | No |
| Family history of psychiatric illnesses (***for Controls***) | Yes | No |
| ***If you checked YES to any of the above questions, STOP. This subject is not eligible!*** | | |

**2. Consent Checklist**

|  | **Yes** | **No** |
| --- | --- | --- |
| Participant passed consent capacity evaluation (***for Cases***) |  |  |
| Participant asked for input from family member (***for Cases***) |  |  |
| A family member has been contacted and has endorsed participation (***for Cases where required***) |  |  |
| Controls have passed the screening questionnaire (***for Controls***) |  |  |
| Participant has signed consent |  |  |
| Participant has been provided with a copy of the consent form |  |  |

***I certify the above is correct and accurate. Therefore, the participant is eligible to the study Global Research Initiative on the Neurophysiology of Schizophrenia.***

| Consenting Staff Name | Signature | Consenting Date |
| --- | --- | --- |
|  |  |  |
| Enrolling Staff Name | Signature | Enrolling Date |
|  |  |  |

**Supplementary Material 4: The Electrode Layout and Channel Assignment for the Customized 64-Channel BrainCap in the GRINS Project**. All electrodes in the cap are Multitrodes with sintered Ag/AgCl sensors that are suitable for sleep EEG recordings. They can be buttoned directly into the cap (total height: 3.5 mm) or attached to the skin with washers (equivalent to double-sided adhesive rings). All electrodes are named and numbered at the sensor end, and numbered at the socket end. EMG1/EMG2 and VEOG are B29 electrodes. A1/A2, LOC/ROC, and EMG1/EMG2 are dropdown electrodes from the cap. The electrodes “61” and “62” are additionally labeled with “REF” at the socket end, and electrode “63” is additionally labeled with “GND” at the socket end for an alternative setup. The cable colors correspond to the figure below. The cables are secured to the cap with nylon threads. Two cable trees exit the cap in a plait-like fashion behind the ears, pointing downwards. The length of the cable trees is approximately 120 cm. The cable length for the individual electrodes REF and GND is 150 cm.


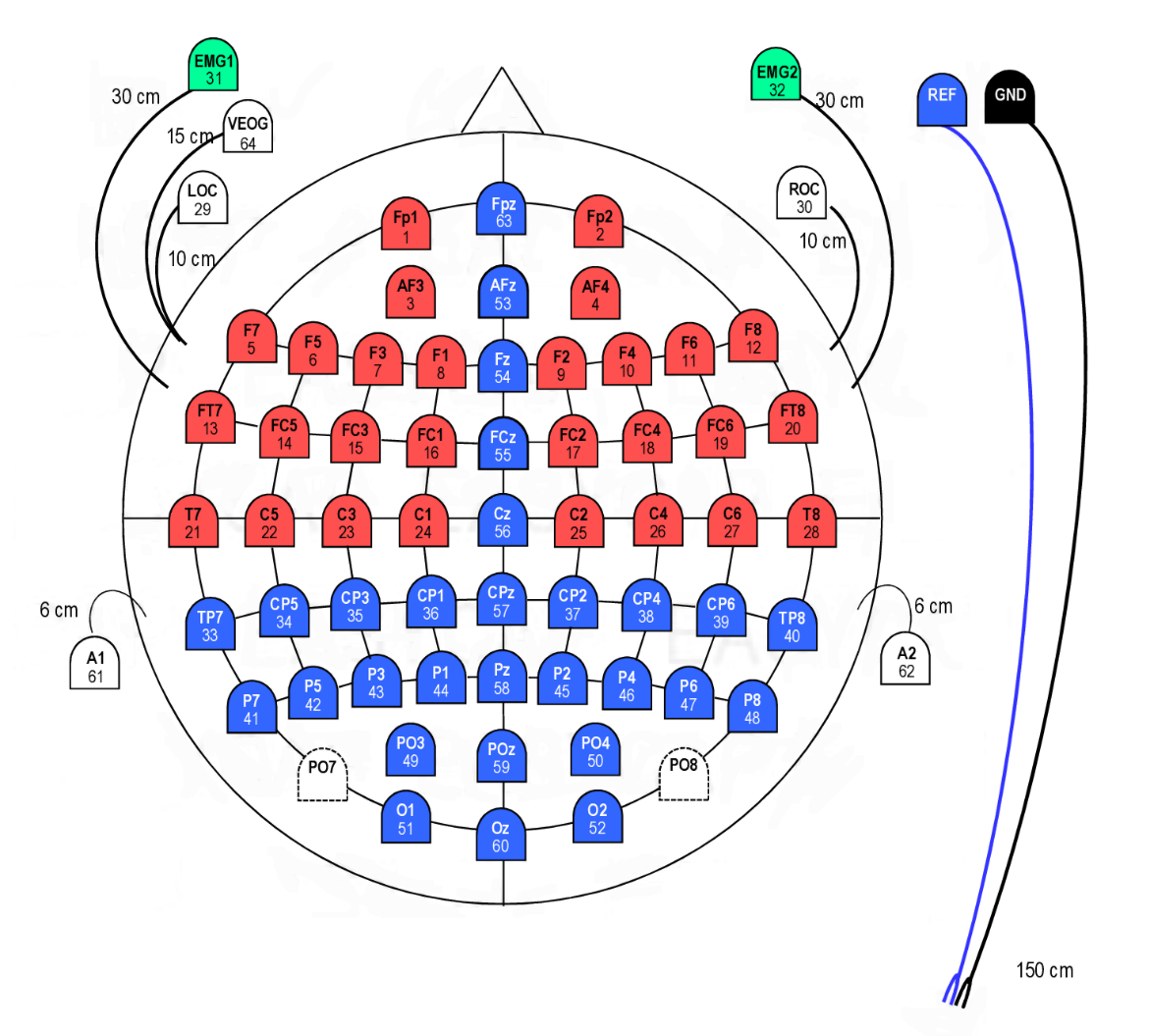


**Supplementary Material 5: Schematics of the Finger-Tapping Test and Motor Sequence Tests.** (A) The finger-tapping test conducted during the introductory interview session, to screen subjects for finger motor dysfunction. (B) A warm-up session is conducted prior to each round of motor sequence tests (MST), requiring participants to repeatedly type '3-4' using their right hands. (C) The first and second rounds of MST conducted during the overnight study session, sharing the same Sequence A, with an interval of around 8 hours of sleep. (D) The third and fourth rounds of MST conducted during the overnight study session, sharing the same Sequence B, with an interval of 10 minutes of rest.


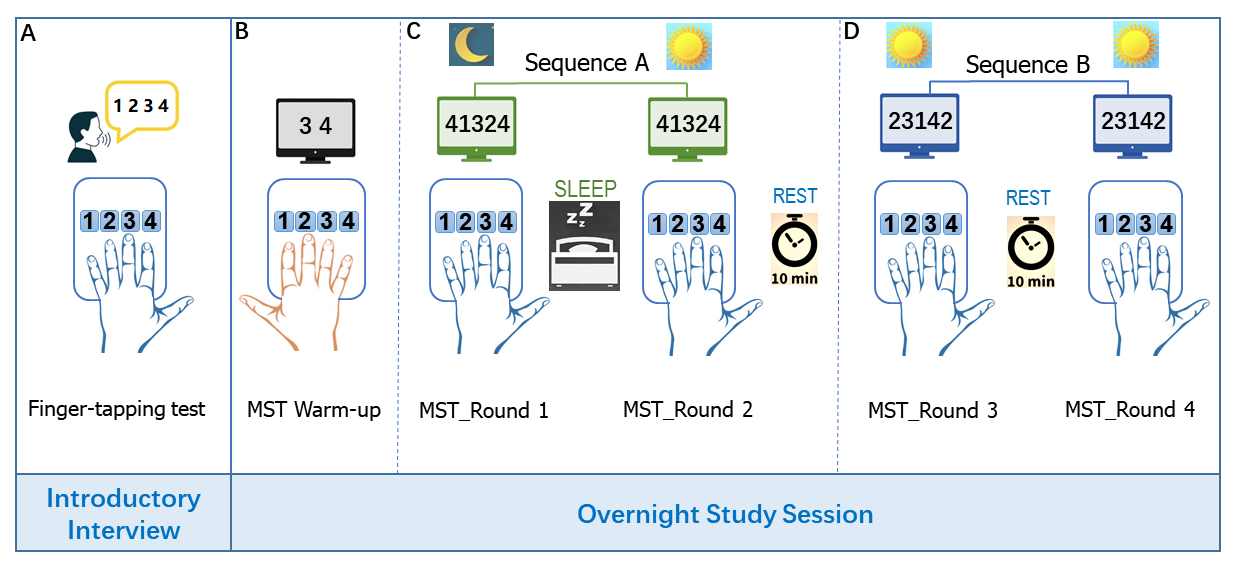

Supplement: Supplementary file 1 — Supplementary Material 1. [file 12888_2024_5882_MOESM1_ESM.docx]
